# Phaeophytin Analogues from *Ligularia knorringiana*

**DOI:** 10.3390/molecules17055219

**Published:** 2012-05-07

**Authors:** Hui Li, Lina Li, Qiusheng Zheng, Chiaki Kuroda, Qi Wang

**Affiliations:** 1 Key Laboratory of Xin Jiang Phytomedicine Resources, School of Pharmacy, Shihezi University, Shihezi 832002, China; Email: xiaohuiyaoshi@163.com (H.L.); 214594367@qq.com (L.L.); johnsonjem@hotmail.com (Q.Z.); 2 Department of Chemistry, Rikkyo University, Nishi-Ikebukuro, Toshima-ku, Tokyo 171-8501, Japan; Email: kuroda5000144@grp.rikkyo.ne.jp

**Keywords:** *Ligularia knorringiana*, phaeophytins, ligulariaphytin A

## Abstract

A new phaeophytin, ligulariaphytin A, together with five known phaeophytins, were isolated from the aerial parts of *Ligularia knorringiana*. The structure of ligulariaphytin A was elucidated as 13^1^-hydroxy-13^1^,13^2^-peroxyphaeophorbide A ethyl ester (**1**), and the five known compounds were identified as 13^2^-hydroxyphaeophorbide A ethyl ester (**2**), 17^3^-ethoxyphaeophorbide A (**3**), phaeophytin B (**4**), phaeophytin A (**5**), and phaeophorbide B ethyl ester (**6**), respectively, based on spectroscopic analysis and by comparison of their spectral data with those reported previously in the literature. All compounds were evaluated for their *in vitro* cytotoxic activities against cultured Hela cell, and were found to show only very weak cytotoxicity.

## 1. Introduction

**Figure 1:**
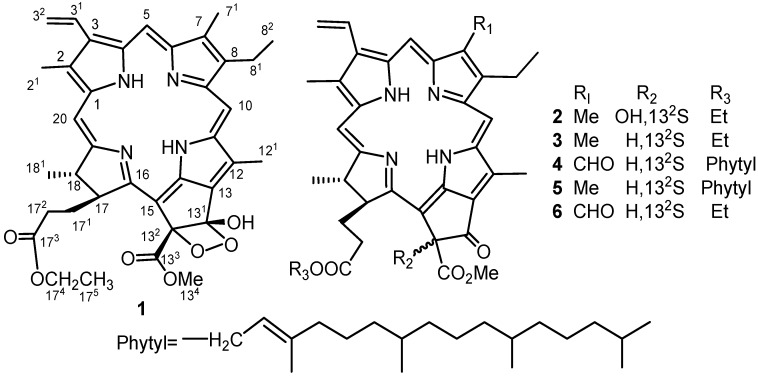
Phaeophytins from *L. knorringiana *aerial parts.

The genus *Ligularia* Cass. is highly diversified in China, consisting of about 110 species [1]. More than 40 species of *Ligularia* have been studied chemically, and more than 300 compounds, including terpenoids, pyrrolizidine alkaloids, flavonoids and benzofuran derivatives, have been isolated, with a variety of biological activities [2,3,4]. One of us and co-workers have been studying the diversity of *Ligularia* by means of analyses of terpenoid composition and neutral DNA sequences and have found that intra-specific diversity is present in many species [5,6]. Although root terpenoid composition has been successfully used as the chemical index, another class of chemicals would be useful to understand the complex diversity. In this study, we focused on phaeophytins, which are found commonly in aerial parts of higher plants. To the best of our knowledge, phaeophytins in *Ligularia* have not been investigated. In this report, we describe isolation of phaeophytins from *L. knorringiana*, whose chemical constituents have not been reported. We isolated a new phaeophytin (**1**) (Figure 1) along with five known phaeophytins, 13^2^-hydroxyphaeophorbide A ethyl ester (**2**) [7], 17^3^-ethoxyphaeophorbide A (**3**) [8], phaeophytin B (**4**) [9], phaeophytin A (**5**) [9], and phaeophorbide B ethyl ester (**6**) [7] from the aerial parts of *L. knorringiana*. The new compound **1** has a rare four-membered peroxide structure.

## 2. Results and Discussion

The aerial parts of *L. knorringiana* were collected in Tianshan mountains, Xinjiang, China, in July 2009, and identified by Prof. Ping Yan, College of Life Sciences, Shihezi University. An EtOH extract of aerial parts of *L. knorringiana* was suspended in H_2_O and partitioned successively with petroleum ether, EtOAc, and *n*-BuOH. Repeated column chromatography of the combined EtOAc and petroleum ether portions on silica gel and RP_18_ gel, followed by preparative TLC, yielded six phaeophytins **1**–**6** (Figure 1).

Compound **1**, trivially named ligulariaphytin A, was obtained as a dark green amorphous solid and was shown to possess the molecular formula C_37_H_40_N_4_O_7_ by HRESIMS (*m/z* 653.2969, [*M*+H]^+^). The IR spectrum showed the presence of an OH group (3,342 cm^−1^), two carbonyls (1,733, 1,702 cm^−1^) and vinyl groups (1,622 cm^−1^). The UV spectrum with absorptions at 670 (2.55), 614 (0.43), 531 (0.577), 500 (0.705), 402 (3.35) nm, along with the corresponding NMR spectra (Table 1) indicated that compound **1** was a phaeophytin [10]. In the ^1^H-NMR spectrum, there were seven methyls at δ 3.27, 3.45, 3.77, 3.90 (each s), 1.72 (t, *J* = 7.6 Hz), 1.07 (t, *J* = 7.2 Hz), and 1.62 (d, *J* = 7.6 Hz); three olefinic singlets at δ 8.72, 9.54, 9.76; one vinyl group at δ 8.03 (dd, *J* = 18.0, 12.0 Hz), 6.32 (br d, *J* = 18.4 Hz), and 6.17 (br d, *J* = 11.2 Hz). In the ^13^C-NMR spectrum, the significant shifts of C(13^1^) (δ 102.1) and C(13^2^) (δ 100.6) indicated that both carbons must be oxygenated, and a C^_^O^_^O^_^C functionality should be present to match the molecular formula [11].

**Table 1:** ^1^H-NMR (400MHz) and ^13^C-NMR (100MHz) data of compound **1** in CDCl_3_.

Pos.	δ_H_ (*J*)	δ_C_		Pos.	δ_H_ (*J*)	δ_C_		Pos.	δ_H_ (*J*)	δ_C_
**1**		141.4 s		**15**		134.6 s		**12^1^**	3.90 (3H, s)	12.3 q
**2**		131.6 s		**16**		166.4 s		**13^1^**		102.1 s
**3**		136.2 s		**17**	4.09 (1H, d, 9.2)	53.8 d		**13^2^**		100.6 s
**4**		136.3 s		**18**	4.47 (1H, q, 7.6)	50.3 d		**13^3^**		171.1 s
**5**	9.54 (1H, s)	99.8 d		**19**		171.3 s		**13^4^**	3.77 (3H, s)	54.4 q
**6**		155.9 s		**20**	8.72 (1H, s)	94.0 d		**17^1^**	1.87 (1H, m)	31.4 t
**7**		136.7 s		**2^1^**	3.45 (3H, s)	12.3 q			2.45 (1H, m)	
**8**		145.7 s		**3^1^**	8.03 (1H, dd, 18.0, 12.0)	129.1 d		**17^2^**	2.18 (1H, m)	32.3 t
**9**		150.2 s		**3^2^**	6.17 (1H, br d, 11.2)	122.9 t			2.55 (1H, m)	
**10**	9.76 (1H, s)	104.3 d			6.32 (1H, br d, 18.4)			**17^3^**		173.5 s
**11**		138.9 s		**7^1^**	3.27 (3H, s)	11.5 q		**17^4^**	3.97 (2H, m)	60.6 t
**12**		131.7 s		**8^1^**	3.74 (2H, m)	19.7 t		**17^5^**	1.07 (3H, t, 7.2)	14.2 q
**13**		111.3 s		**8^2^**	1.72 (3H, t, 7.6)	17.8 q		**18^1^**	1.62 (3H, d, 7.6)	22.4 q
**14**		161.2 s								

**Figure 2:**
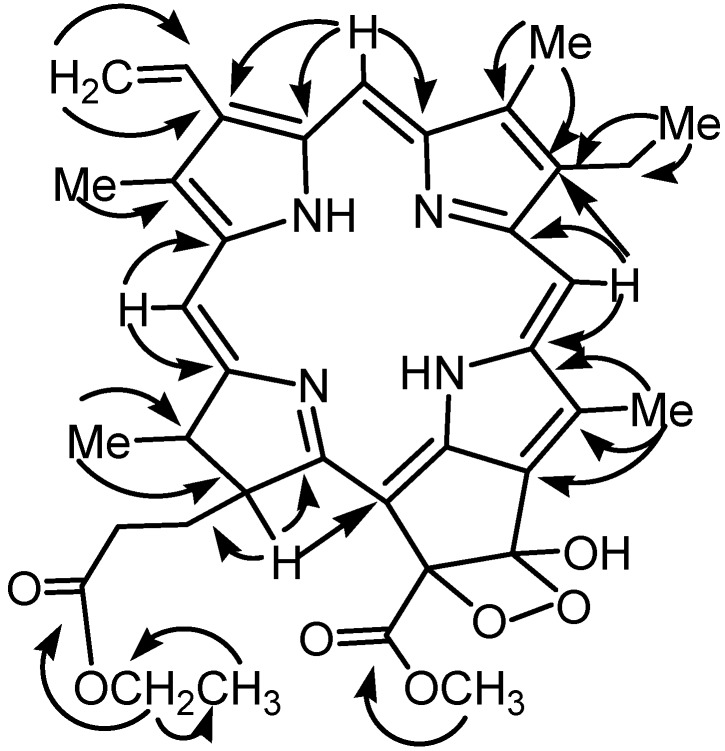
Key HMBC (→) correlations of ligulariaphytin A (**1**).

Compound **1** differed from a known compound bidenphytin B [11] in the position at C-17^3^, where the phytyl ester group in bidenphytin B was replaced by an ethyl ester in **1**, which was shown in the NMR spectra [δ_H_ 3.97 (2H, m), 1.07 (3H, t, *J* = 7.2); δ_C_ 60.6 (CH_2_), 14.2 (CH_3_)]. In the HMBC spectrum of **1** (Figure 2), the long range correlations of the H-17^4^ (δ 3.97) with C-17^3^ (δ 173.5) and C-17^5^ (δ 14.2); the H-17^5^ (δ 1.07) with C-17^4^) (δ 60.6) further confirmed the location of ethyl group. The signal for H-17 (δ 4.09, 1H, d, *J* = 9.2 Hz), indicated that H-17 and H-18 were *trans*-oriented [12]. The absolute configurations at both C-13^1^ and C-13^2^ were analogously assigned as (*S*) by spectroscopic correlation with bidenphytin B [10,11]. Accordingly, the structure of compound **1** was identified as 13^1^-hydroxy-13^1^,13^2^-peroxyphaeophorbide a ethyl ester. Although it is plausible that compound **1** is an artifact produced during isolation with ethanol, we think the compound is a natural product because three known natural ethyl esters **2**, **3**, and **6** were also isolated [7,8]. Compound **1** may be an addition product of **3** with molecular oxygen.

Previous works revealed that phaeophytins possess potent antioxidant activities [13] and cytotoxic activities [14]. All compounds isolated and prepared in this study were therefore assayed for *in vitro* cytotoxicity against the Hela cell line by the methylthiazolyltetrazolium (MTT) assay [15]. Compounds **1** and **2** showed only very weak cytotoxicity, with IC_50_ values of 80, 90 µg/mL, respectively. 

The results indicate that various phaeophytins are present in *Ligularia*, suggesting that the compounds may be a useful index in the study of chemical diversity. Compound **1** has a rare four-membered peroxide-containing phaeophytin and such compound has only been isolated from the Asteraceae once before [11]. The analogues, bidenphytin A and B have been previously isolated from *Biden pilosa* (Asteraceae) [11]. These data suggest that phaeophytins are a useful index to compare various plants chemically.

## 3. Experimental

### 3.1. Plant Material

*Ligularia knorringiana* Pojark. (Asteraceae) is distribution in Northwest China. The aerial parts of *L. knorringiana* were collected in Tianshan mountains, Xinjiang Province, China, in July, 2009. The plant was identified by Prof. Ping Yan, College of Life Sciences, Shihezi University, and a voucher specimen (WQ-LK-09-1) has been deposited in the Herbarium, Department of Pharmacognosy, School of Pharmacy, Shihezi University, Xinjiang, China.

### 3.2. General

Melting points were measured on an XT-4 micro-melting point apparatus and are uncorrected. Optical rotations were recorded on a JASCO P-1020 polarimeter at room temperature. UV spectra were measured on a Shimadzu UV-260 spectrophotometer in absolute MeOH. IR spectra were recorded on an Avatar 360 FT-IR ESP spectrometer in KBr. A Bruker Daltonics APEXШ 7.0 TESLA FTMS mass spectrometer for HRESIMS. NMR spectra were recorded on a Varian XTIPC-400 spectrometer (400 MHz for ^1^H and 100 MHz for ^13^C). Analytical and preparative TLC were run on silica gel plates (GF_254_, Yantai Institute of Chemical Technology, Yantai, China). Spots were observed under UV light and visualized by spraying with 10% H_2_SO_4_, followed by heating. Column chromatography (CC) was performed on silica gel (200–300 mesh and 300–400 mesh; Qingdao Marine Chemical Factory, Qingdao, China) and Lichroprep RP_18_ gel (40–60 μm, Merck, Darmstadt, Germany).

### 3.3. Extraction and Isolation

The air-dried, aerial parts (6 kg) of *L. knorringiana *were extracted exhaustively with 95% aq. EtOH (3 × 50 L) at r.t. The EtOH extract was concentrated *in vacuo* to yield a semi-solid (800 g), which was suspended in H_2_O (800 mL), and extracted successively with PE (3 × 1,500 mL), CHCl_3_ (3 × 1,500 mL), EtOAc (3 × 1,500 mL) and *n*-butanol (3 × 1,500 mL). The organic phases were concentrated to yield residues with 160 g, 50 g, 18 g, and 50 g, respectively. The combined PE and CHCl_3_ extracts (200 g) was subjected to CC (6 kg of SiO_2_; PE/acetone gradient) to afford ten fractions (*Fr*.*1*–*Fr*.*10*). *Fr*.*7*, eluted with PE/acetone 5:1, was subjected to repeated CC (SiO_2_; CHCl_3_/MeOH 300:1) to afford **6** (8 mg). *Fr*.*8*, eluted with PE/acetone 3:1, was subjected to repeated CC (SiO_2_; CHCl_3_/MeOH 400:1-200:1) to afford **3** (25 mg). *Fr*.*9*, eluted with PE/acetone 2:1, was subjected to repeated CC (SiO_2_; CHCl_3_/MeOH 400:1-200:1), and then with PTLC (CHCl_3_/MeOH 70:1) to afford **2** (50 mg), with PTLC (CHCl_3_/MeOH 140:1) to afford **4** (30 mg), and **5 **(37 mg). *Fr*.*10*, eluted with PE/acetone 1:1, was subjected to repeated CC (SiO_2_; PE/acetone 3:1), and then followed by PTLC (PE/EtOAc 1:1) to afford **1** (4 mg).

*Ligulariaphytin A* (**1**): dark green amorphous solid; 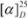 +862 (*c* 0.06, CHCl_3_); IR ν_max_ 3342, 2964, 2869, 1733, 1702, 1622, 1452 cm^−1^; UV (CHCl_3_): 670 (2.55), 614 (0.43), 531 (0.577), 500 (0.705), 402 (3.35) nm; ^1^H-NMR (CDCl_3_, 400 MHz) and ^13^C-NMR (CDCl_3_, 100 MHz) data see Table 1; TOF-MS: 653.2969 ([*M+*H]^+^, C_37_H_40_N_4_O_7_^+^; calc. 653.2975).

## 4. Conclusions

As a part of our chemical investigation on the aerial parts of *Ligularia knorringiana*, a new phaeophytin, ligulariaphytin A, together with five known phaeophytins, were isolated and their structures elucidated by spectroscopic methods including 2D-NMR techniques. The results indicate that various phaeophytins are present in *Ligularia*, suggesting that the compounds may be a useful index in the study of chemical diversity. Compounds **1** and **2** showed very weak cytotoxicity against cultured HeLa cella.

## Supplementary Materials

Supplementary materials can be accessed at: http://www.mdpi.com/1420-3049/17/5/5219/s1.
