# Supplementary material for: Phaeophytin Analogues from Ligularia knorringiana
Source: Molecules. 2012 May 7;17(5):5219–24. doi: 10.3390/molecules17055219 (PMC6268028; doi:10.3390/molecules17055219)

Supporting Materials

1. ^{1}H-NMR spectrum (400 MHz, $CDCl_3$) of compound **1**

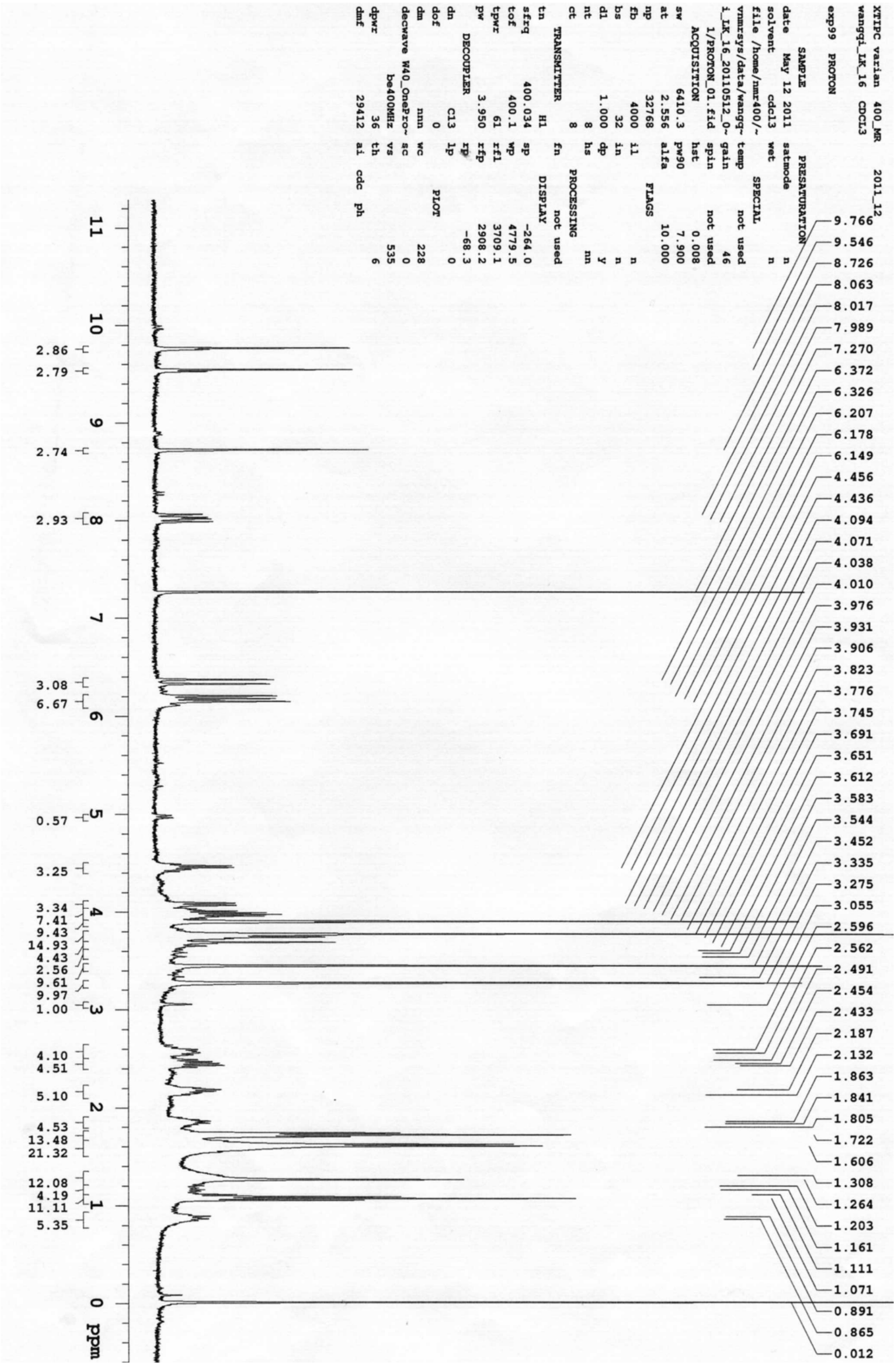

2. ^{13}C-NMR spectrum (100 MHz, $CDCl_3$) of compound **1**

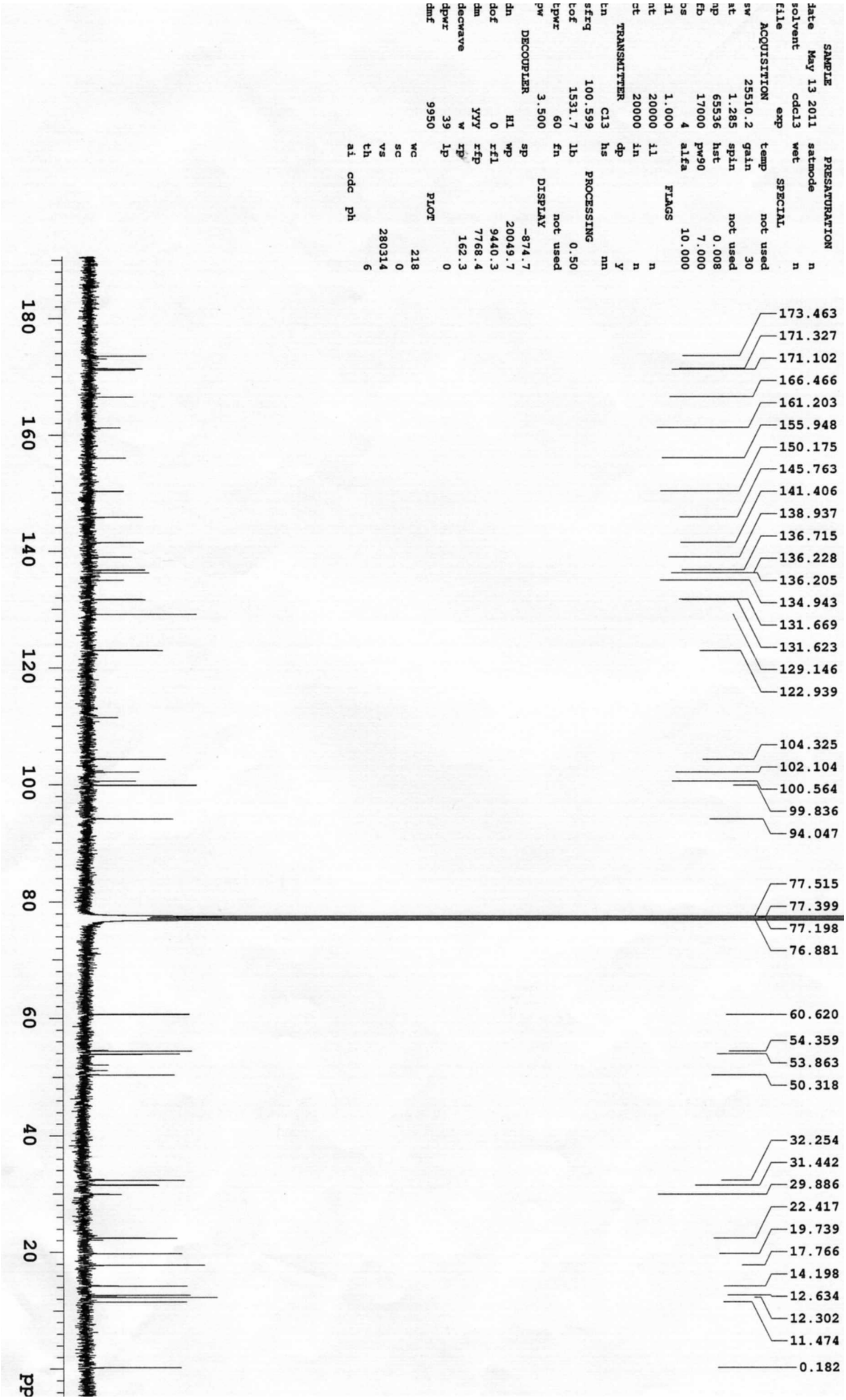

3. HMBC spectrum of compound **1**

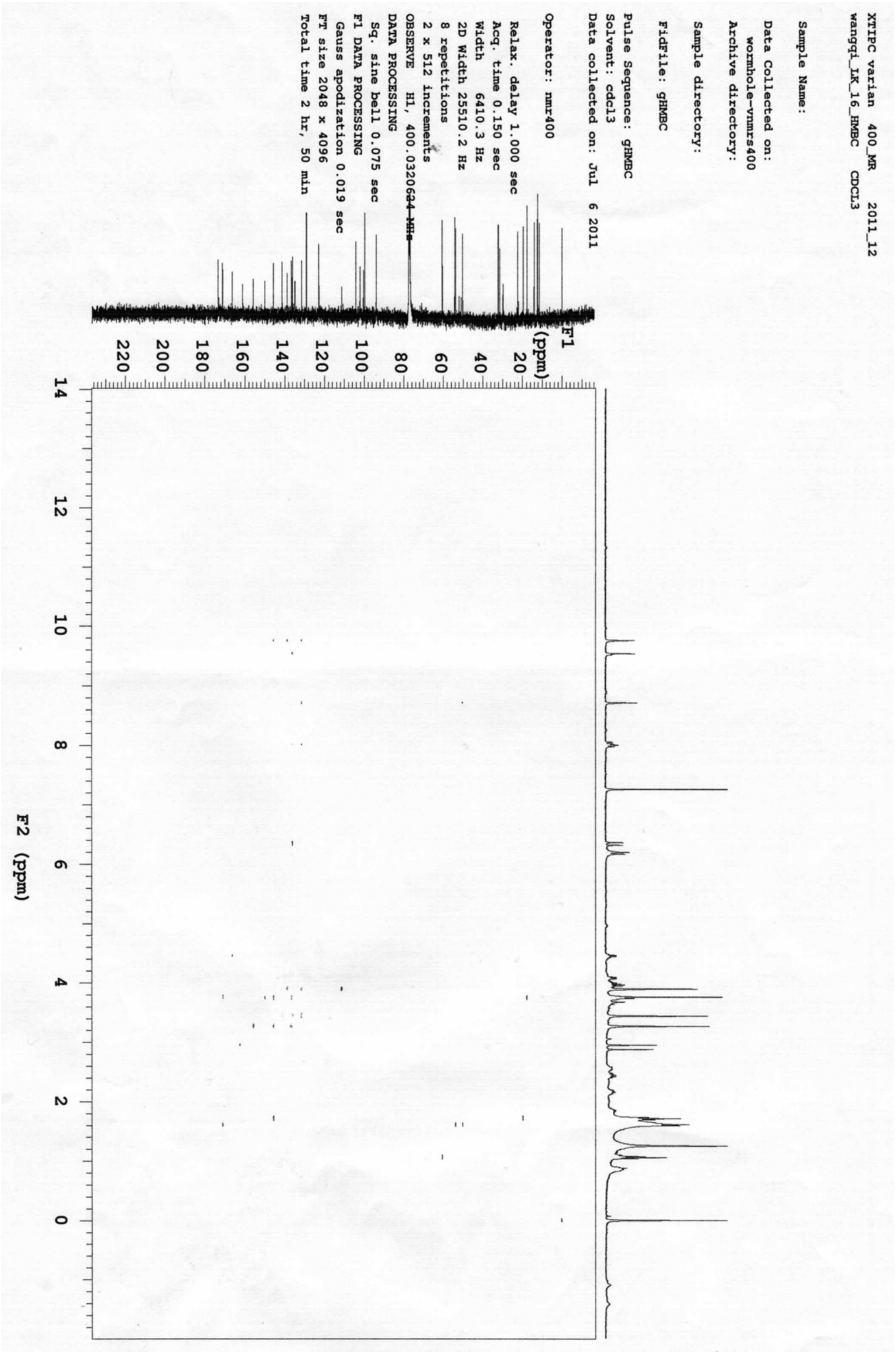

Supplement: Supplementary file 1 [file molecules-17-05219-s001.pdf]
